# Reversing the pipeline: a ‘human-first’ multi-omics approach to cardiovascular discovery

**DOI:** 10.1038/s12276-026-01835-8

**Published:** 2026-09-09

**Authors:** Elchanan Parnasa, Yair Rokach, Suzan Abedat, Offer Amir, Iddo Z. Ben-Dov, Rabea Asleh

**Affiliations:** 1 Cardiovascular Research Center, Heart Institute, Hadassah Medical Center, Faculty of Medicine, Hebrew University of Jerusalem, Jerusalem, Israel; 2 Department of Internal Medicine B, Faculty of Medicine, Hebrew University of Jerusalem, Jerusalem, Israel

**Keywords:** Translational research, Cardiovascular diseases

## Abstract

Despite significant biomedical advances, cardiovascular disease remains a leading cause of global mortality. The traditional ‘bench-to-bedside’ research pipeline, which heavily relies on animal models to guide clinical translation, is often hampered by species-specific physiological differences, leading to high rates of clinical trial failures. In this Review, we advocate for a paradigm shift toward a ‘human-first’ discovery approach. We explore how integrating human cardiac tissue resources obtained from biopsies, surgical explants and autopsies with high-resolution multi-omics technologies (including transcriptomics, proteomics and metabolomics) is transforming our understanding of complex heart diseases. We highlight recent breakthroughs in heart failure, ischemic heart disease, arrhythmias, myocarditis and valvular stenosis achieved through direct profiling of human samples. Finally, we propose a ‘bedside-to-bench-and-back’ strategy, in which therapeutic targets are identified in human tissues and then validated in animal models to accelerate the development of precise, clinically relevant cardiovascular treatments.

**Figure:**
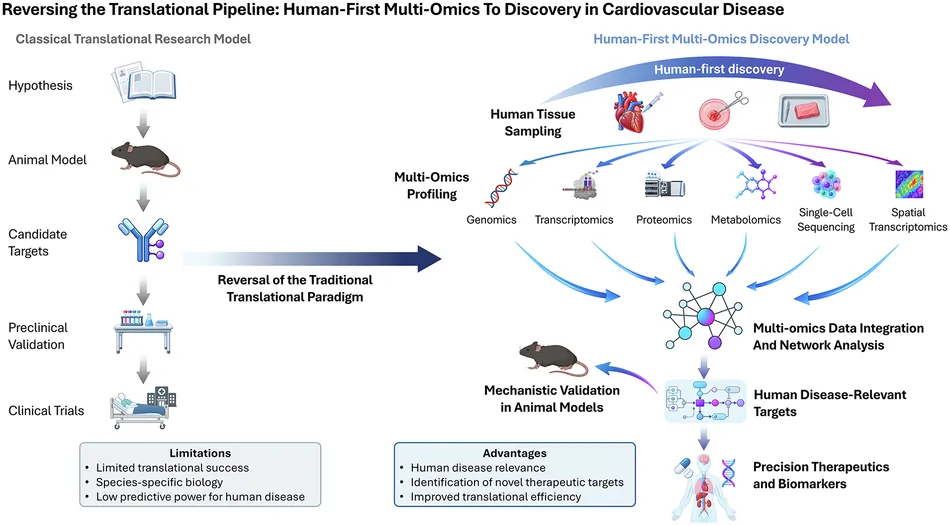

## Reversing the cardiovascular discovery pipeline: from animal-first translation to human-first molecular discovery

The graphical abstract contrasts the conventional bench-to-bedside model with the human-first framework proposed in this Review. In the traditional pipeline, hypotheses are usually generated from literature and preclinical animal models, followed by mechanistic studies, therapeutic testing and eventual clinical translation. Although this approach has produced major advances, it is limited by species-specific differences in cardiovascular physiology and by the inability of many animal models to fully recapitulate complex human disease. In the proposed human-first model, discovery begins with clinically derived human cardiovascular tissues, including biopsies, surgical specimens, explanted hearts, donor tissues and autopsy samples. These samples are analyzed using high-resolution molecular technologies such as transcriptomics, proteomics, metabolomics, epigenomics, single-cell sequencing and spatial omics. Candidate mechanisms identified directly in human tissue are then prioritized for experimental validation in animal models, organoids, engineered tissues or other perturbable systems. The goal is not to eliminate animal models, but to reposition them as causal-validation platforms downstream of human molecular discovery. This iterative bedside-to-bench-and-back strategy is intended to improve translational fidelity, identify clinically relevant mechanisms and accelerate the advancement of precision cardiovascular medicine.

This summary was initially drafted using artificial intelligence, then revised and fact-checked by the author.

## Introduction

Cardiovascular disease remains a leading cause of global mortality despite major advances in biomedical research and clinical care. Historically, several cardiovascular breakthroughs stemmed from direct human observation. Although modern biomedical research has largely adopted an animal-first approach, high-resolution molecular technologies now enable direct analysis of human tissues to identify disease mechanisms and therapeutic targets, which can then be validated in experimental systems.

**Fig. 1:**
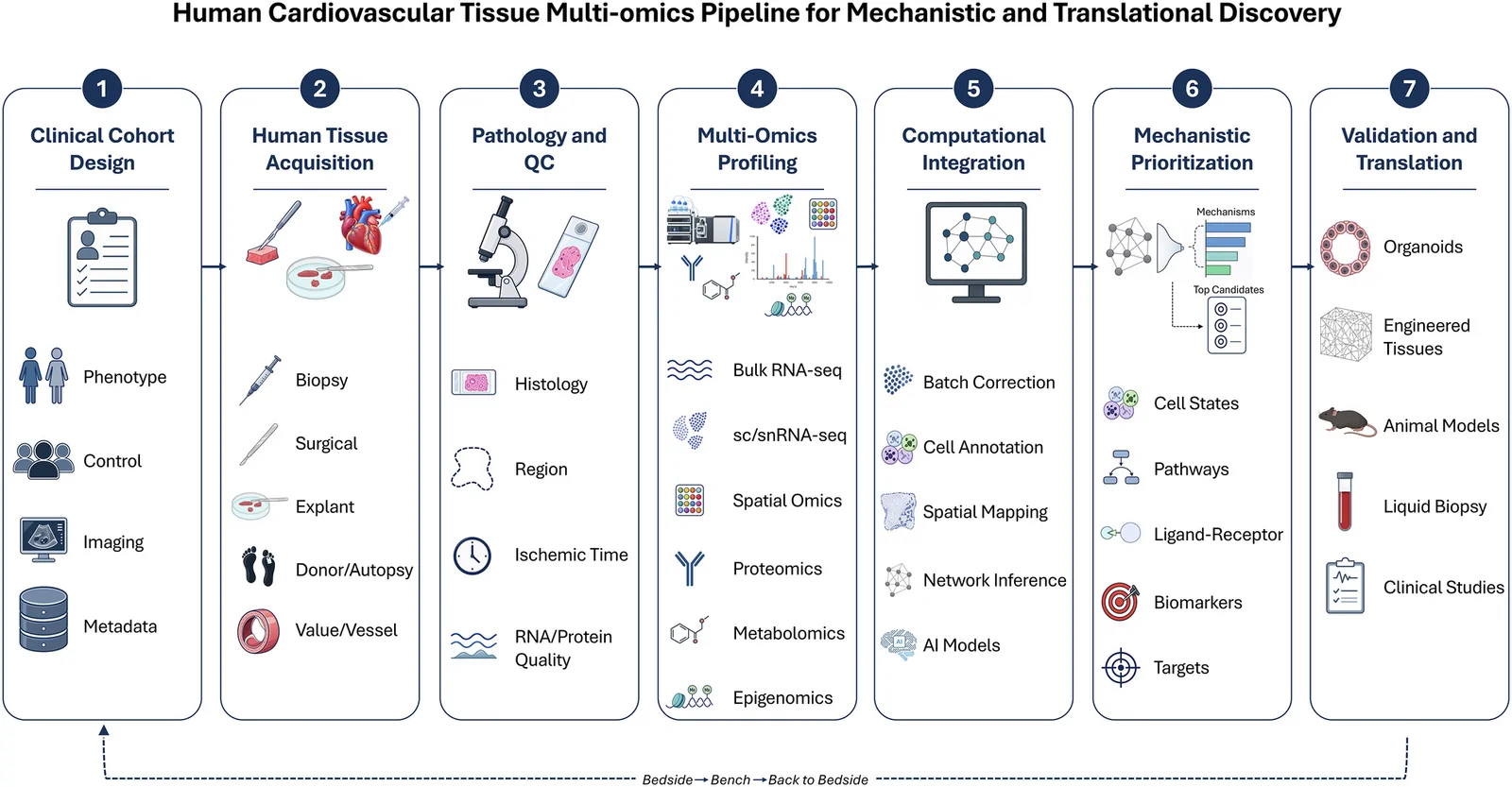
Human cardiovascular tissue multi-omics pipeline for mechanistic and translational discovery. The figure illustrates a structured human-first workflow for cardiovascular multi-omics studies. The pipeline begins with clinical cohort design, including disease phenotyping, matched comparison groups, imaging, medications, comorbidities, longitudinal outcomes and standardized metadata collection. Human cardiovascular tissues are then obtained from endomyocardial biopsies, surgical specimens, explanted hearts, donor hearts, autopsy material, vascular tissue or valvular tissue. Each sample undergoes pathology review and quality control, including anatomical sampling-site documentation, histology, ischemic time, fixation or freezing method, RNA integrity, proteomic quality metrics and assessment of tissue heterogeneity. Molecular profiling may include bulk transcriptomics, single-cell or single-nucleus RNA sequencing, spatial transcriptomics, spatial proteomics, proteomics, metabolomics, epigenomics and targetsed validation assays. Computational integration then harmonizes multilayered datasets using batch correction, cell-state annotation, spatial mapping, network inference, machine learning and biological foundation models. Candidate mechanisms are prioritized on the basis of reproducible disease-associated cell states, pathways, ligand–receptor interactions, spatial niches, circulating biomarkers and druggable targets. Finally, selected mechanisms are tested using orthogonal human cohorts, organoids, engineered heart tissues, animal models, liquid biopsy platforms and ultimately clinical studies. This stepwise workflow operationalizes the bedside-to-bench-and-back framework proposed in this Review.

In practical terms, a human-first strategy relies on clinically derived cardiac tissues, including biopsies, surgical specimens, explanted and donor hearts, and autopsy samples, together with genomics, proteomics, metabolomics and spatial technologies (Fig. 1). As emphasized by Fayyaz et al., such resources can identify therapeutic targets directly from human disease biology before experimental validation^1–3^.

Human cardiac tissue sources offer distinct opportunities and limitations. Endomyocardial biopsies allow direct interrogation of diseased myocardium but are limited to specific clinical indications, particularly transplant rejection surveillance, and are constrained by small sample sizes, limited availability of controls and myocardial spatial heterogeneity^1,4,5^. Explanted hearts and surgical specimens provide larger tissue amounts but may be affected by advanced disease, cardioplegia, perioperative ischemia, donor brain death and treatment exposure. Autopsy material can provide access to control or early-disease tissue and has supported large-scale proteomic studies, although post-mortem interval and tissue preservation require careful adjustment^6–8^.

Multi-omics technologies are also essential for defining normal human cardiac biology. The human heart atlas by Litviňuková et al., based on single-cell and single-nucleus RNA sequencing across cardiac chambers, revealed chamber-specific cell states, cellular diversity and rare subpopulations, which now serve as a reference for interpreting human heart disease^9^.

This Review examines how human cardiovascular tissues, integrated with transcriptomic, proteomic, metabolomic, single-cell and spatial approaches, can identify molecular drivers of cardiovascular disease (Table 1). We focus on clinically derived samples from heart failure, ischemic heart disease, arrhythmias, myocarditis, cardio-oncology, amyloidosis and valvular disease. Selected landmark examples are summarized in Table 2, with a broader evidence base in Supplementary Table 1. The central premise is not to replace animal models but to reposition them as causal-validation platforms downstream of human tissue discovery, thereby creating a bedside-to-bench-and-back framework.

**Table 1:** Multi-omics and complementary molecular techniques for human cardiac tissue analysis.

Technique	Advantages	Limitations
Genomics(1)	Enables genome-wide analysis of DNA/RNA Identifies mutations and regulatory variants High-throughput and scalable	Lacks post-transcriptional/protein insight Requires computational analysis Functional impact not always clear
Proteomics(2)	Measures protein abundance and modifications Reflects cellular function and pathways Supports biomarker discovery	Technically complex and variable Limited detection of low-abundance proteins Lower throughput than genomics
Metabolomics(3)	Captures metabolic activity and biochemical state Sensitive to physiological/environmental changes Complements omics data	High variability between samples Limited metabolite databases Requires specialized platforms
Western blot(4)	Detects specific proteins with size info Cost-effective and widely used Useful for validation	Low throughput and semi-quantitative Antibody specificity critical Limited reproducibility
Immunohistochemistry (IHC)(5)	Localizes proteins in tissue context Clinically relevant; used in diagnostics Reveals spatial expression patterns	Antibody-dependent specificity Semi-quantitative; subjective interpretation Background noise possible
Histological staining(6)	Visualizes tissue morphology and structure Rapid and cost-effective Common in pathology and research	No molecular specificity Cannot assess gene/protein levels Often requires complementary techniques

*GWAS* genome-wide association study, *LC–MS* liquid chromatography-mass spectrometry, *NMR* nuclear magnetic resonance, *RNA-seq* RNA sequencing, *SNP* single-nucleotide polymorphism.

This table summarizes key omics layers, detailing their molecular targets, analytical methods, applications in cardiovascular disease, and the advantages and limitations of each approach.

**Table 2:** Selected landmark human-tissue multi-omics studies and translational milestones in cardiovascular disease.

Disease area	Omics approach	Human tissue resource	Mechanistic or translational milestone	References
Normal human cardiac biology	Single-cell and single-nucleus transcriptomics	Multichamber adult human heart tissue	Defined the cellular diversity of the adult human heart, including chamber-specific cell states and rare populations, establishing a reference atlas for disease interpretation.	^9^
HFpEF	Myocardial transcriptomics	Human myocardial biopsies	Identified HFpEF-specific transcriptional programs distinct from HFrEF, supporting the concept that HFpEF is not simply a milder form of systolic failure but a molecularly distinct syndrome.	^2^
HFpEF	Myocardial metabolomics	Human LV myocardial tissue and plasma	Revealed myocardial metabolic inflexibility, including altered fatty acid oxidation, TCA-cycle intermediates, ketone metabolism and branched-chain amino-acid metabolism.	^3^
Heart failure recovery	Transcriptomics and phosphoproteomics	LVAD-treated human myocardium	Identified molecular signatures that distinguish myocardial recovery from persistent failure, linking mechanical unloading to cell-cycle and extracellular matrix remodeling pathways.	^24^
Cardiomyopathy	Single-nucleus transcriptomics	Human DCM, HCM and control hearts	Identified disease-associated fibroblast states and candidate regulators of myofibroblast differentiation, connecting human cell-state discovery with functional validation.	^33^
Heart failure fibrosis	Single-cell multi-omics and ligand–receptor analysis	Human failing myocardium	Revealed immune–fibroblast communication involving IL-1, TNF and TGF-β signaling; subsequent experimental validation supported this axis as a causal fibrotic pathway.	^27^
Ischemic heart disease	Spatial multi-omics	Human post-myocardial infarction tissue	Mapped infarct and border-zone remodeling in spatial context, identifying region-specific fibroblast, myeloid, endothelial and cardiomyocyte programs.	^36^
Ischemic heart disease	Spatial transcriptomics and transcriptomics	Human right atrial tissue from ischemic heart disease	Identified microvascular inflammation and metabolic reprogramming associated with ischemic remodeling and the development of heart failure.	^37^
Atherosclerosis	Single-cell immune profiling	Human carotid plaques	Defined immune-cell diversity in plaques and linked symptomatic disease to activated T ell and macrophage programs associated with instability.	^40^
Atherosclerosis	Single-cell and spatial transcriptomics	Human carotid plaques	Identified the microvasculature as an inflammatory niche characterized by endothelial activation, lymphocyte trafficking and smooth muscle cell transition.	^44^
Cardiac conduction system	Spatially resolved multi-omics	Human cardiac conduction tissue	Revealed specialized cardiac niches, including fibroblast–glial interactions and immune-cell microenvironments, advancing understanding of human cardiac electro-anatomy.	^47^
AF	Single-nucleus multi-omics	Human left atrial tissue	Identified cardiomyocyte androgen receptor signaling and disease-associated fibroblast states, linking sex-related epidemiology to tissue-level remodeling in AF.	^50^
Myocarditis	Single-cell and spatial immune profiling	Human myocardial tissue and biopsies	Identified cause-specific immune and stromal programs in viral, vaccine-associated and ICI-related myocarditis, supporting mechanism-based disease stratification.	^54–57^
Cardio-oncology	Proteomics	Human cardiac tissue and anthracycline-exposed cardiac microtissues	Identified mitochondrial, translation, intracellular signaling and NF-κB-related protein networks implicated in anthracycline cardiotoxicity.	^63^
Cardiac amyloidosis	Mass spectrometry-based proteomics	Human endomyocardial biopsies and amyloid-containing myocardium	Enabled precise molecular subtyping of ATTR, AL and other amyloid forms, directly supporting clinically actionable diagnosis.	^65–69^
Post-infarction amyloid remodeling	Integrated multi-omics	Human and experimental cardiac tissue	Identified stromal extracellular vesicle signaling as a driver of amyloid deposition after myocardial infarction, and pharmacological inhibition prevented amyloid accumulation in models.	^68^
Calcific aortic stenosis	Proteomics and histology	Human calcific aortic valve tissue	Identified apoC-III enrichment and linked lipoprotein-associated inflammatory and mitochondrial pathways to valvular calcification.	^73^
Calcific aortic stenosis	Spatial proteogenomics and systems biology	Human calcific aortic valve tissue	Revealed layer-specific molecular remodeling, inflammatory and fibrotic networks, and candidate therapeutic pathways in calcific valve disease.	^74^
Calcific aortic stenosis	Single-cell and proteomic profiling	Human calcific aortic valve tissue	Identified a disease-driver valvular interstitial cell population with osteogenic potential and candidate regulators of mineralization.	^81^

*ECM* extracellular matrix, *HFrEF* heart failure with reduced ejection fraction, *LV* left ventricular.

This table summarizes representative paradigm-shifting studies that illustrate how human cardiovascular tissues, combined with advanced molecular profiling, have changed mechanistic understanding, enabled target discovery, or supported translational development. The comprehensive evidence table is provided as Supplementary Table 1.

## The use of human cardiac tissue in heart failure research

Heart failure with preserved ejection fraction (HFpEF) illustrates why human myocardial tissue is essential for cardiovascular discovery. HFpEF is clinically heterogeneous, strongly influenced by age, sex, obesity, renal dysfunction, vascular disease and systemic inflammation, and remains difficult to treat because its mechanisms are incompletely resolved^10^.

**Fig. 2:**
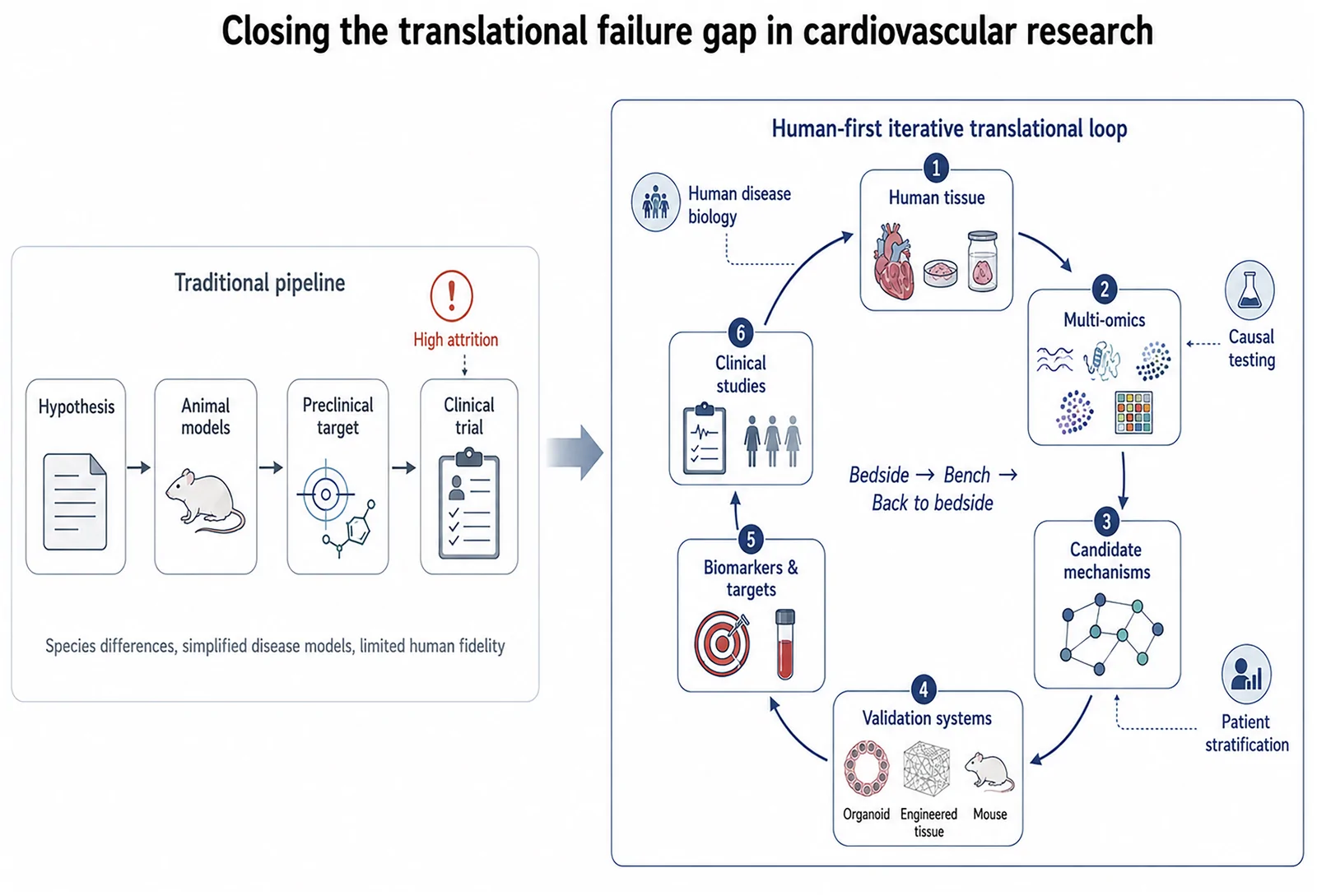
Closing the translational failure gap in cardiovascular research. The figure contrasts the conventional animal-first translational pipeline with the proposed human-first iterative framework. In the traditional model, hypotheses are generated and tested primarily in animal models, leading to preclinical target nomination before clinical trials. Although this approach remains essential for mechanistic testing, it is limited by species-specific physiology, simplified disease modeling and an incomplete representation of human cardiovascular heterogeneity, which contribute to high clinical attrition. The human-first framework begins with direct molecular interrogation of diseased human cardiovascular tissues, followed by multi-omics profiling to identify candidate mechanisms present in the clinical disease state. These candidates are then tested in perturbable validation systems, including organoids, engineered tissues and animal models, to establish causality and therapeutic tractability. Validated mechanisms can subsequently be translated into circulating biomarkers, therapeutic targets, patient stratification tools and clinical studies. The loop from clinical studies back to human tissue discovery emphasizes an iterative bedside-to-bench-and-back model in which human disease biology guides experimental validation and translational development.

Existing animal models reproduce selected components of HFpEF but rarely capture its full comorbidity-driven human phenotype, including sex-specific and systemic features^1,11,12^. Animal models remain indispensable for controlled mechanistic testing and therapeutic validation (Fig. 2). This limitation is illustrated by the NO–sGC–cGMP pathway: despite favorable experimental data, clinical trials targeting this axis in HFpEF failed to demonstrate meaningful benefit^13–15^. This supports a workflow in which human myocardial profiling identifies disease-relevant pathways, and experimental systems subsequently test causality.

### Multi-omics technologies for mechanistic discovery in heart failure

Unlike conventional biomarkers such as BNP or NT-proBNP, integrated multi-omics can interrogate the molecular layers that drive heart failure, including genomic variation, transcriptional programs, epigenetic regulation, protein remodeling and metabolic state (Table 1 and Fig. 1). These approaches can link inflammation, fibrosis, metabolism, vascular dysfunction and remodeling to disease classification, risk prediction, therapeutic target discovery and trial enrichment^16–19^. Their value is greatest when applied directly to human cardiac tissue, as peripheral blood or peripheral blood mononuclear cell-derived data may not fully capture local myocardial biology.

To address these challenges, studies based on human tissue have provided physiologically relevant insights into heart failure. For example, single-cell analysis of 21,422 cells from human hearts, including normal, failing and left ventricular assist device (LVAD)-supported hearts, revealed the central role of cardiomyocytes in contractility and metabolism, along with critical interactions with noncardiomyocytes within complex cellular networks^20,21^. These findings clarify the molecular effects of LVAD support and emphasize the importance of human-derived tissues in modeling disease states that are hard to replicate in animal systems^21^.

The value of advanced molecular approaches is shown by Chaffin et al., who used single-nucleus RNA sequencing of human heart samples from dilated cardiomyopathy (DCM), hypertrophic cardiomyopathy (HCM) and control hearts to identify a unique fibroblast subgroup in failing hearts. Functional validation using CRISPR-mediated gene disruption reduced myofibroblast differentiation in response to transforming growth factor-β1 (TGF-β1), highlighting disease-specific cellular mechanisms and potential therapeutic targets.

Advanced multi-omics analyses of human cardiac tissue enable the identification of disease mechanisms, therapeutic targets and biomarkers of progression^19^. For example, Tan et al. showed that epigenetic changes, including early shifts in CpG methylation, predict progression to overt heart failure in DCM, highlighting their potential as diagnostic and therapeutic targets^19,22^.

### Translating myocardial multi-omics findings into circulating biomarkers

A key translational goal is to link tissue-defined myocardial programs to circulating biomarkers that can be measured repeatedly in clinical practice. Rather than treating blood proteins or metabolites as isolated statistical associations, they should be anchored to tissue-level processes, including fibrosis, inflammation, hypertrophy, metabolic stress, vascular remodeling or cardiomyocyte injury. For example, Perry et al. linked circulating proteins to echocardiographic phenotypes and myocardial transcriptional signatures, suggesting that blood-based proteomic signals can reflect tissue-level heart failure biology^23^. This tissue-to-blood strategy helps complete the bedside-to-bench-and-back loop by translating invasive myocardial discoveries into scalable tools for diagnosis, risk stratification, longitudinal monitoring, trial enrichment and assessment of therapeutic response.

Multi-omics approaches also offer insights into the molecular effects of heart failure therapies. Drakos et al. analyzed myocardial tissue from LVAD-treated patients using transcriptomic and phosphoproteomic profiling, identifying distinct molecular signatures that differentiate failing from nonfailing myocardium and responders from nonresponders^24^. These findings highlight pathways related to cell cycle regulation and extracellular matrix remodeling that underlie functional recovery^24^.

Human myocardial studies in HFpEF demonstrate that tissue omics can elucidate disease mechanisms that circulating biomarkers alone cannot capture. Single-cell analyses identified cell-type-specific transcriptional changes in cardiomyocytes, fibroblasts, endothelial cells and immune cells, including inflammatory and fibrotic programs^25^. Matched tissue–blood analyses showed only a weak correlation between myocardial *NPPB* expression and circulating NT-proBNP, underscoring the influence of renal function, age and body mass index on biomarker interpretation^26^. Metabolomic and transcriptomic profiling further revealed impaired branched-chain amino acid metabolism, altered tricarboxylic acid (TCA) cycle intermediates, disrupted fatty acid oxidation, increased inflammatory and fibrotic signaling, and reduced expression of mitochondrial and oxidative metabolism genes^2,3,23^.

To better characterize the remodeling processes that eventually lead to heart failure, single-cell RNA sequencing of human failing myocardium identified activated fibroblast and immune cell populations and uncovered pro-fibrotic signaling networks, including interleukin (IL)-1, tumor necrosis factor (TNF) and TGF-β-mediated crosstalk^27^. Ligand–receptor analysis further clarified immune–stromal interactions, while validation in murine models demonstrated that targeting these pathways reduced fibrosis and preserved cardiac function^27^. These findings highlight the importance of human tissue-based discovery combined with validation using animal models.

Analysis of myocardial tissue from patients with advanced heart failure revealed a link between inflammation and mitochondrial dysfunction, especially in ischemic cardiomyopathy. Ischemic cardiomyopathy samples exhibited increased inflammatory infiltration and impaired mitochondrial function, with lower oxidative capacity and citrate synthase activity, while fibrosis was not clearly associated with mitochondrial performance^28^. These findings indicate that inflammatory signatures contribute to bioenergetic deficits and could be targets for immunomodulatory therapy.

To unravel the cellular complexity behind inflammation and fibrosis in heart failure, Rao et al. used single-cell and T cell receptor sequencing of human myocardial tissue to identify immune–stromal interactions that drive these processes in heart failure^29^. Their analysis revealed activated myofibroblast populations, T cell remodeling toward pro-inflammatory and exhausted states, and macrophage–endothelial interactions involving a CXCL8–DARC signaling axis that promotes immune cell recruitment. These findings establish a coordinated multicellular network underlying chronic inflammation and fibrosis in human heart failure^29^.

Human myocardial multi-omics has also clarified mechanisms underlying genetic cardiomyopathies and sudden cardiac death. Proteomic profiling in HCM identified dysregulation of Ras–MAPK signaling, protein degradation, angiogenesis, metabolism, inflammation and extracellular matrix remodeling, linking molecular signatures to disease severity^30^. Epigenomic analyses showed that noncoding regulatory variation influences cardiac gene expression through enhancer H3K27ac quantitative trait loci and long-range mechanisms^31^. Postmortem myocardial studies further identified cardiomyopathy-related variants in cases with primary myocardial fibrosis, supporting the value of molecular autopsy for family risk assessment^32^. Together, these studies demonstrate how direct human tissue analysis can reveal disease mechanisms and stratification markers that may be missed by animal models alone^1^.

Collectively, human myocardial multi-omics reframes heart failure as a multicellular disease ecosystem rather than a purely cardiomyocyte-centered disorder. Single-cell, single-nucleus, proteomic, metabolomic and spatial approaches identify coordinated remodeling across cardiomyocytes, fibroblasts, endothelial cells, immune cells, the extracellular matrix and metabolic pathways^2,3,20,21,23,26,27,29,33^. This is particularly relevant in HFpEF, where ejection fraction and circulating biomarkers do not adequately distinguish inflammatory-fibrotic, metabolic, microvascular or systemic disease endotypes. Human tissue discovery can therefore refine disease classification, nominate mechanism-based therapeutic targets and prioritize pathways for experimental validation.

## The use of human cardiac tissue in ischemic heart disease research

The investigation of ischemic heart disease has made significant progress through the analysis of human cardiac tissue, especially in revealing complex pathobiological interactions, including the relationship between microbial presence and innate immune activation within the myocardium^34,35^.

Studies of human cardiac tissue have uncovered complex cellular and immune mechanisms involved in ischemic heart disease. Using single-cell and spatial multi-omics techniques, Kuppe et al. created a detailed map of post-ischemic remodeling, highlighting important roles for fibroblasts, myeloid cells, endothelial cells and cardiomyocytes^36^. Meanwhile, analyses of surgical myocardial samples identified microbial signatures associated with Toll-like receptor activation and chronic inflammation^34,35^. Together, these findings show that direct molecular profiling of human tissue uncovers previously unknown cellular interactions and inflammatory pathways that drive ischemic remodeling.

A key advantage of spatial omics in ischemic heart disease is that it preserves the anatomical context in which inflammatory and fibrotic programs occur. Bulk RNA sequencing can identify differentially expressed pathways in the ischemic myocardium, but it cannot determine whether these signals arise from the infarct core, border zone, viable remote myocardium, vascular structures, infiltrating immune aggregates or extracellular-matrix-rich scar regions. Similarly, circulating biomarkers may reflect systemic myocardial injury or inflammation, but they cannot localize the tissue compartment where the molecular process is occurring. Spatial transcriptomics and spatial multi-omics address this limitation by linking gene expression programs to histological architecture, thereby enabling investigators to map cardiomyocyte stress, fibroblast activation, endothelial dysfunction, immune cell recruitment and matrix remodeling within intact myocardial tissue^34,36,37^.

Human ischemic and atherosclerotic tissues have revealed disease mechanisms at cellular resolution. Transcriptomic and spatial profiling of right atrial tissue after ischemia identified microvascular inflammation and metabolic reprogramming linked to heart failure^37^. In coronary and carotid plaques, single-cell, spatial and multimodal analyses identified inflammatory macrophage states, activated and exhausted T cell subsets, endothelial-to-mesenchymal transition, smooth muscle-derived fibromyocyte phenotypes and enrichment of genetic risk within macrophage, endothelial and smooth muscle compartments^38–41^. These studies shift the view of atherosclerosis from a vessel-wall lipid-storage model to a spatially organized immune–vascular–stromal disease process.

Human tissue studies have also mapped coronary artery innervation, identifying sympathetic nerve networks around major coronary vessels that are difficult to model in animals with comparable anatomical precision^42^.

The importance of human tissue research in cardiovascular disease goes beyond cardiac tissues to include human vascular tissues. For example, Shami et al. demonstrated that GITR expression in carotid plaques is associated with plaque instability and cerebrovascular events, whereas circulating sGITR levels are associated with increased cardiovascular risk^43^. Combining spatial transcriptomics with single-cell RNA sequencing, studies of human carotid plaques identified the microvasculature as a key inflammatory niche characterized by endothelial activation, chemokine signaling and immune cell recruitment^44^. These analyses further revealed lymphocyte trafficking via ACKR1⁺ endothelial cells, smooth muscle cell transition into fibromyocytes, and complex cell–cell interactions that drive atherogenesis^44^.

Taken together, these studies show that human tissue-based multi-omics redefines ischemic heart disease as a multicellular and spatially organized process rather than a cardiomyocyte-focused pathology. By integrating single-cell and spatial analyses, this approach uncovers previously unrecognized cellular interactions and inflammatory niches that drive post-ischemic remodeling and plaque instability, emphasizing the important role of human tissue in mechanistic discovery. Clinically, this reframes ischemic disease as a spatially compartmentalized inflammatory-stromal process and supports the development of imaging, biomarker, and cell-state-guided therapeutic strategies.

Beyond structural and ischemic heart diseases, direct analysis of human cardiac tissue has also greatly improved the understanding of cardiac electrophysiology and arrhythmogenesis.

## The use of human cardiac tissue in electrophysiology research

The importance of human cardiac tissue in electrophysiological research has been recognized since the 1960s and 1970s, when early studies using human surgical samples established fundamental differences in atrial and ventricular action potentials, providing key insights into cardiac electrophysiology^45,46^. A recent study by Kanemaru et al. on human cardiac conduction tissue yielded important findings. Using multi-omics profiling of human cardiac conduction tissue, they uncovered complex cellular interactions, including fibroblast–glial relationships and immune niches involving plasma cells, offering new insights into cardiac electro-anatomy and its immune modulation^47^. These findings emphasize mechanisms that are not easily observed in animal models. Another study, conducted by Vyas et al., investigated the molecular mechanisms of atrial fibrillation (AF) and highlighted the role of epicardial adipose tissue (EAT) in disease development. Analysis of human cardiac tissue revealed an enrichment of tissue-resident memory T cells within EAT, linking local immune activation to inflammatory and fibrotic remodeling and to increased susceptibility^48^.

As previously mentioned, AF remains a heterogeneous and incompletely understood arrhythmia, with significant variation in clinical presentation and underlying mechanisms^49^. Importantly, differences between species in cardiac electrophysiology reduce the relevance of animal models, which often fail to replicate sustained human AF, highlighting the need for human tissue-based research^49^.

Recently, the application of single-nucleus multi-omics analysis has offered unprecedented insights into the cellular and molecular mechanisms driving AF^50^. This approach revealed differential gene expression patterns in cardiomyocytes of patients with persistent AF, notably involving androgen receptor signaling pathways. These findings may provide a molecular explanation for the well-documented higher prevalence of AF in men compared with women^50^. In addition, the study identified a distinct subpopulation of fibroblasts that appears to be actively involved in the pathogenesis of persistent AF. This subpopulation may contribute to atrial fibrosis, thereby facilitating the maintenance and progression of the arrhythmia^50^. Importantly, this research was conducted using human cardiac tissue samples obtained from the left atrium of patients undergoing cardiac surgery for various clinical indications^50^.

Together, these studies indicate that arrhythmogenesis in humans cannot be fully understood by ion channel biology alone. Human tissue multi-omics has revealed that conduction and rhythm disorders arise from specialized cellular niches that include cardiomyocytes, fibroblasts, glial-like cells, immune cells and inflammatory programs associated with EAT^47,48,50^. In AF, the identification of cardiomyocyte androgen receptor signaling and disease-associated fibroblast states provides a mechanistic bridge between epidemiological observations, such as sex differences in AF prevalence, and tissue-level remodeling that promotes atrial fibrosis and electrical instability^49,50^. These findings have direct translational relevance, suggesting that future antiarrhythmic strategies may need to target the inflammatory-stromal and adipose tissue microenvironment, rather than focusing exclusively on electrophysiological ion channel modulation.

In addition to structural and electrophysiological disorders, inflammatory cardiac diseases also provide a significant context in which analysis of human myocardial tissue has yielded essential mechanistic insights.

## The use of human cardiac tissue in myocarditis research

Myocarditis is a diverse inflammatory disease with multiple causes that remains difficult to diagnose and treat, often resulting in severe outcomes such as death or progression to DCM. Analysis of human cardiac tissue has provided essential insights into its underlying mechanisms and potential therapeutic targets. Autopsy-based analyses of human cardiac tissue have revealed interactions between viral infection and genetic predisposition in myocarditis, linking viruses such as human herpesvirus 6, parvovirus B19 and Epstein–Barr virus with underlying cardiomyopathic phenotypes^51^. These findings suggest that myocarditis-related sudden death may reflect latent genetic susceptibility, with implications for cascade screening. Simultaneously, detection of viral genomes in myocardial tissue using polymerase chain reaction-based approaches offers clinically relevant diagnostic and prognostic tools^52^.

Another important area of research has investigated the relationship between immune checkpoint inhibitor (ICI) use and myocarditis. Using human cardiac tissue, infiltration of cytotoxic CD8⁺/PD-1⁺ T cells and granzyme B activity were observed within the myocardium^53^. Spatial transcriptomics further identified T cell and macrophage infiltration, granulocyte accumulation and elevated cytokines (IL-10, IL-15 and IL-13), thereby uncovering key inflammatory pathways and potential therapeutic targets^54^. These findings highlight mechanisms that are not adequately captured in current animal models. Another study investigating ICI-induced myocarditis, using single-cell RNA sequencing of human myocardial biopsies in ICI-associated myocarditis, identified key stromal and endothelial drivers of inflammation, including CXCL9^+^ fibroblasts with interferon-responsive signatures and ACKR1^+^ endothelial cells mediating immune cell recruitment^55^. These findings highlight a non-immune cellular network orchestrating myocardial inflammation.

The study of myocarditis development using advanced molecular profiling of human heart tissue has gained significant momentum since the start of the COVID-19 pandemic. Both severe acute respiratory syndrome coronavirus 2 (SARS-CoV-2) infection and messenger RNA (mRNA)-based vaccination have been associated with causing inflammatory heart injury, highlighting the need for in-depth mechanistic research. In one such study, single-nucleus transcriptomic profiling of human heart tissue identified distinct immune signatures across different causes of myocarditis, including post-SARS-CoV-2 infection and mRNA vaccination^56^. Post-infectious myocarditis was characterized by interferon-γ responses and CD8⁺/NK cell activity, whereas vaccine-related myocarditis exhibited increased IL-16/IL-18 signaling and CD4⁺ T cell activity, along with common myeloid activation and endothelial dysfunction^56^. These results reveal that different causes of myocarditis involve unique molecular processes that drive myocardial inflammation.

To improve understanding of ICI-associated myocarditis, Blum et al. conducted a multicompartment single-cell and proteomic analysis of human cardiac tissue, blood and tumor samples, identifying a unique inflammatory signature in ICI-associated myocarditis characterized by cytotoxic CD8⁺ T cells, dendritic cells and activated fibroblasts^53,57^. T cell receptor sequencing showed myocardium-expanded T cell clones that were not shared with tumor tissue, indicating antigen specificity independent of antitumor immunity and linking specific clones to fatal outcomes^57^. These findings underscore a divergence between antitumor responses and immune-mediated cardiac damage.

Together, these studies show that human tissue-based multi-omics characterizes myocarditis as a range of cause-specific immune and stromal responses. By combining single-cell and molecular profiling, this method uncovers different disease pathways in viral, ICI-related and vaccine-associated myocarditis, aiding the development of targeted treatments.

Another rapidly advancing field where human cardiac tissue analysis has proven especially valuable is cardio-oncology.

## The use of human cardiac tissue in cardio-oncology research

Cardio-oncology, a quickly growing field examining the impact of cancer and its treatments on the heart, has increasingly benefited from studies involving human cardiac tissue. As cancer survival rates improve, treatment-related cardiotoxicity has become a significant clinical challenge, prompting efforts to uncover underlying molecular mechanisms and identify predictive biomarkers^58^. For example, Udin et al. analyzed postmortem human cardiac tissue from patients with cancer and showed that structural changes, including collagen disorganization and decreased cardiomyocyte nuclear density, are associated with higher BNP levels and echocardiographic dysfunction, thereby linking tissue remodeling to clinical cardiac impairment^59^.

Additional studies have focused on identifying biomarkers that predict cardiotoxicity during cancer treatment. Kushnareva et al. demonstrated that myocardial PD-1/PD-L1 signaling is linked to cardiac dysfunction, with PD-L1 levels inversely related to left ventricular ejection fraction and directly related to left ventricular end-diastolic volume, indicating that immune checkpoint imbalance may contribute to the risk of cardiotoxicity during cancer therapy^60^.

Multi-omics approaches have also been used to identify biomarkers of cardiovascular risk in cancer survivors. For example, metabolomic profiling has identified elevated levels of trimethylamine N-oxide, a gut microbiome-derived metabolite associated with adverse cardiovascular outcomes, highlighting the role of therapy-induced microbiome alterations in cardiotoxicity among cancer patients^61,62^.

Multi-omics approaches have also been used to study chemotherapy-induced cardiotoxicity. Nguyen et al. applied proteomic profiling of human cardiac tissue and microtissues exposed to anthracyclines, which identified protein networks linked to mitochondrial dysfunction, protein translation and intracellular signaling, with key mediators such as HSPA5, BAG3 and SH3BGRL implicated in cardiotoxicity^63^.

Network analysis further identified a core set of proteins involved in mitochondrial homeostasis and nuclear factor-κB (NF-κB) signaling, reinforcing their role as mediators and potential therapeutic targets in anthracycline-induced cardiotoxicity^63^.

Beyond treatment-related cardiotoxicity, studies of human myocardial tissue have also yielded important insights into infiltrative cardiomyopathies such as cardiac amyloidosis.

## The use of human cardiac tissue in amyloidosis research

Despite therapeutic advances, the mechanisms underlying tissue-specific injury in cardiac amyloidosis remain only partially understood, making analysis of human myocardial tissue essential to elucidate the molecular composition of amyloid deposits and their impact on cardiac structure and function^64^.

Proteomic profiling of human cardiac tissue revealed distinct molecular signatures in transthyretin amyloidosis (ATTR) versus light-chain amyloidosis (AL), with ATTR enriched in complement and contractile proteins, while AL was characterized by keratin-related pathways, reflecting different cellular responses^65^. Additional findings, including activation of endoplasmic reticulum stress pathways such as QSOX1, suggest compensatory mechanisms in ATTR^65^.

A separate study analyzing myocardial biopsies revealed stage-dependent molecular remodeling, with advanced AL amyloidosis associated with coagulation, extracellular matrix, hypoxia and endocytic pathways, while ATTR showed enrichment of complement and immunoglobulin-related processes^66^. Furthermore, human myocardial studies have connected amyloidosis to atrial remodeling and arrhythmogenesis, with increased telocyte numbers and macrophage infiltration indicating active structural and inflammatory changes^67^.

To explore the pathological role of stromal-derived extracellular vesicles in post-infarction amyloid formation, Cimini et al. conducted integrated multi-omics analyses that revealed that stromal-derived extracellular vesicles can drive amyloid deposition following myocardial infarction, with podoplanin⁺ stromal cells releasing serum amyloid A3, which activates macrophages via TLR2 signaling and promotes fibril formation^68^. Pharmacologic inhibition of this pathway prevented amyloid accumulation and preserved cardiac function, identifying a potential therapeutic target^68^.

Mass spectrometry-based proteomics enables precise molecular subtyping of cardiac amyloidosis, differentiating among ATTR, AL and other amyloid types while identifying proteins involved in fibril stability and disease progression. This enhances diagnostic accuracy^69^.

Human tissue-based multi-omics approaches have also revolutionized the understanding of valvular heart diseases, especially calcific aortic stenosis.

## The use of human cardiac tissue in aortic valve stenosis research

Aortic valve stenosis (AS) is the most common valvular heart disease and mainly results from progressive degenerative calcification of the valve leaflets. There has been an increasing focus on understanding the molecular mechanisms underlying disease development. Multi-omics approaches that integrate genomic, transcriptomic, proteomic and metabolomic data from human valve tissue have become powerful tools for studying calcific AS^70,71^. By integrating insights across molecular layers, this method reveals regulatory mechanisms and interdependent pathways that might otherwise remain hidden. Using systems biology for target selection can aid in the development of new pharmacological therapies as alternatives to valve replacement procedures^67^.

One of the earliest proteomic studies of human aortic valves identified altered proteins involved in inflammation, oxidative stress and extracellular matrix remodeling in calcified valves, offering early molecular insight into AS pathogenesis^72^. To explore the role of lipoprotein particles in the development of calcific aortic valve disease, Schlotter et al. used proteomic profiling and immunohistochemistry of human valve tissue, showing that several apolipoproteins, particularly apolipoprotein C-III (apoC-III), are enriched in calcified valve regions and promote calcific transformation of valvular interstitial cells through inflammatory and mitochondrial pathways^73^.

A multimodal systems biology analysis of human aortic valves revealed spatially distinct molecular signatures across valve layers, including activation of inflammatory pathways, transformation of valvular interstitial cells into smooth muscle-like cells, and ongoing calcification, especially within fibrosa-derived cell populations^74^.

Additional work identified endothelial dysfunction as an early factor in calcific valve disease, with the loss of PALMD disrupting nuclear export signaling and cytoskeletal organization, thereby promoting a procalcific transcriptional phenotype even in morphologically intact valve tissue^75^. Another study aimed to elucidate the valvular pathophysiology of AS, showed that proteomic analysis of myocardial tissue from patients with different AS phenotypes revealed significant alterations in the cardiac ryanodine receptor (RyR2), particularly in AS-associated heart failure with reduced ejection fraction. This suggests that molecular differences between AS subtypes may influence disease progression and therapeutic responses^76^.

Comparative vesiculomic analysis of extracellular vesicles from carotid plaques and stenotic aortic valves revealed distinct molecular signatures, indicating that calcific AS is a biologically unique disease process rather than merely a manifestation of systemic atherosclerosis^77^. Given the clinical and molecular connections between atherosclerosis and AS, metabolomic profiling of myocardial tissue from patients with AS-related left ventricular hypertrophy revealed a unique metabolic signature marked by long-chain acyl-carnitine buildup, impaired fatty acid oxidation and suppression of the PPAR-α regulatory pathway^78^.

Interestingly, multi-omics analyses have indicated mechanistic connections between AS and amyloidosis, with molecular profiling of calcified valves revealing signatures like those of ATTR and suggesting that amyloid-related pathways may contribute to valvular degeneration^79^. Beyond data acquisition, multi-omics enables pathway-level analysis of functional links between proteins and metabolites, revealing interconnected networks in calcific AS involving metabolic dysregulation, hypoxia signaling and purine metabolism, with ABCA8 emerging as a central regulatory hub^80^. Multi-omics approaches also differentiate between clinically similar but molecularly distinct forms of aortic stenosis. Comparative transcriptomic and metabolomic analyses of tricuspid versus bicuspid calcific valves revealed subtype-specific differences in mitochondrial dysfunction, oxidative stress and extracellular matrix remodeling, with greater upregulation of matrix remodeling genes, including *MMP9*, *MMP11*, *MMP12* and *MMP13*, in bicuspid disease, consistent with its more aggressive clinical course^78,81–83^.

Single-cell multi-omics studies have further clarified the cellular drivers of calcific aortic valve disease by identifying a rare fibrosa-resident valvular interstitial cell subset with strong osteogenic potential, while functional analyses implicated MAOA and CTHRC1 as regulators of valve mineralization^79,84^. Beyond identifying cellular drivers of disease, omics-based approaches have also uncovered myocardial effects of AS, with proteomic profiling of atrial and ventricular tissues revealing changes associated with hypertrophy, fibrosis, mitochondrial dysfunction and impaired myocardial perfusion^85,86^. Taken together, these findings show that multi-omics analyses of human valvular and myocardial tissues reveal the cellular and molecular mechanisms underlying calcific aortic stenosis and highlight the value of human tissue-based research for identifying clinically relevant therapeutic targets.

## From human discovery to experimental validation and clinical translation

Several examples in this Review illustrate how human tissue-based molecular discovery can move beyond descriptive profiling toward experimental validation and clinical relevance. In heart failure, single-cell analysis of the human failing myocardium identified activated fibroblast populations and inflammatory ligand–receptor networks involving IL-1, TNF and TGF-β signaling^27^. Importantly, these human-derived observations were tested in experimental models, where disruption of IL-1β signaling attenuated pathogenic fibroblast activation, reduced myocardial fibrosis and improved cardiac function^27^. This example demonstrates how human myocardial single-cell data can identify intercellular pathways for subsequent in vivo causal evaluation.

A second example is cardiac amyloidosis, where human myocardial proteomics has direct diagnostic and translational implications. Proteomic profiling of human cardiac tissue has distinguished ATTR from AL amyloidosis, revealed subtype-specific molecular remodeling and enabled mass spectrometry-based amyloid typing in endomyocardial biopsies^65–69^. In addition, integrated multi-omics analyses identified stromal-derived extracellular vesicle signaling as a contributor to post-infarction amyloid deposition, with pharmacological inhibition of this pathway preventing amyloid accumulation and preserving cardiac function in experimental models^68^. Thus, in amyloidosis, human tissue proteomics supports both clinically actionable molecular classification and mechanistic target discovery.

Similar translational logic is emerging in cardio-oncology and valvular heart disease. In anthracycline-induced cardiotoxicity, proteomic analyses integrating human cardiac tissue and cardiac microtissues identified mitochondrial dysfunction, protein translation, intracellular signaling and NF-κB-related protein networks as candidate mechanisms and therapeutic nodes^63^. In calcific aortic stenosis, human valve proteomics and spatially resolved molecular analyses have identified apoC-III enrichment, inflammatory and mitochondrial pathways, and PALMD-related endothelial dysfunction as candidate disease mechanisms that can be functionally tested in experimental systems^73,75^. Collectively, these examples support the central premise of the bedside-to-bench-and-back model: human tissues should serve as the primary discovery platform, while animal models, organoids, microtissues and other experimental systems should be used to test causality, refine mechanisms and prioritize clinically relevant interventions.

## Future directions and current challenges in human tissue multi-omics

Despite rapid advances in human tissue-based multi-omics research, several challenges still need to be addressed to fully realize the translational potential of these methods. A significant limitation is the scarcity of well-annotated human cardiac tissues, particularly from suitable control groups and early disease stages. In addition, the inherent spatial and cellular heterogeneity of the human heart complicates sampling strategies and data analysis. For this reason, future human cardiovascular multi-omics studies should increasingly pair molecular profiling with preserved tissue architecture, using spatial transcriptomics, spatial proteomics, multiplex imaging and histology-guided sampling to preserve regional information central to myocardial inflammation, fibrosis, ischemic injury and vascular remodeling.

### Human tissue access, control selection and intermediate validation systems

A major practical limitation of human cardiovascular tissue multi-omics is the scarcity of appropriate control tissue. Unlike blood-based studies, in which samples can be collected from healthy volunteers, myocardial tissue from truly healthy individuals is rarely available. Most available ‘control’ specimens are obtained from donor hearts unsuitable for transplantation, surgical discard tissue, autopsy material or nondiseased regions of hearts affected by other conditions. Each of these sources carries potential confounders. Donor hearts may be influenced by brain death, catecholamine exposure, hemodynamic instability, intensive care treatment and cold ischemia. Autopsy samples may be affected by agonal state, post-mortem interval, tissue degradation and variable preservation. Surgical samples may be influenced by cardioplegia, anesthesia, perioperative ischemia, comorbidities, medications and sampling location. Therefore, control selection in human cardiac tissue research should not be treated as a simple binary distinction between ‘healthy’ and ‘diseased’ myocardium but as a structured design problem requiring careful phenotyping and transparent reporting.

Several strategies can help the field overcome these barriers. First, future studies should prioritize standardized tissue acquisition protocols that document the anatomical sampling site, ischemic time, fixation or freezing method, RNA integrity, proteomic quality metrics, histological assessment, medication exposure, comorbidities and clinical trajectory. Second, studies should avoid pooling biologically distinct control sources without conducting sensitivity analyses. Whenever possible, donor hearts, autopsy samples, surgical specimens and adjacent non-involved tissue should be analyzed as separate control strata or included with covariate adjustment. Third, multiregion sampling should be encouraged, particularly in diseases such as heart failure, myocardial infarction, myocarditis, amyloidosis and valvular heart disease, where remodeling is spatially heterogeneous. Fourth, molecular data should be paired with histology, imaging and clinical metadata to distinguish disease-specific signals from tissue-source artifacts.

Large-scale human tissue initiatives offer an important partial solution to this problem. The Genotype-Tissue Expression (GTEx) project established a major resource linking human tissue samples with gene expression and genetic variation, providing a reference for tissue-specific regulatory architecture across the body^87,88^. Although GTEx is not a disease-specific cardiovascular biobank and relies largely on post-mortem tissues, it provides an essential baseline for interpreting tissue-specific expression and regulatory variation. Similarly, the Human Cell Atlas and human heart atlas efforts have begun to define normal cellular diversity and chamber-specific cell states in the adult human heart^9^. The Human BioMolecular Atlas Program further extends this concept by developing spatially resolved, single-cell-level maps of human tissues, providing a framework for understanding molecular organization within intact tissue architecture^89^. Together, these resources can help define reference states against which diseased cardiovascular tissues can be compared.

However, general tissue atlases cannot replace disease-focused cardiovascular biobanks. The next step should be to develop harmonized, multicenter cardiovascular tissue biobanks that integrate biopsies, explanted hearts, surgical specimens, autopsy samples, blood and plasma, imaging, histology and longitudinal clinical outcomes. Such biobanks should include standardized consent procedures, harmonized tissue-processing protocols, shared metadata standards and reproducible pipelines for transcriptomics, proteomics, metabolomics, epigenomics and spatial analysis. Importantly, they should also include clinically relevant comparison groups, such as nonfailing donor hearts, early-stage disease samples, advanced disease samples and treatment-exposed tissues. This structure would allow investigators to separate disease biology from confounding effects related to tissue source, disease stage, therapy and sample handling.

Human induced pluripotent stem (iPS) cell-derived systems can serve as an intermediate experimental bridge between human tissue discovery and animal validation. Patient-specific iPS cell-derived cardiomyocytes, cardiac organoids, cardioids and engineered heart tissues can model selected aspects of human cardiac biology in a controlled, perturbable environment. Self-assembling human heart organoids and heart-forming organoids recapitulate key features of early cardiac development and multicellular cardiac organization^90,91^. Cardioids derived from human pluripotent stem cells further demonstrate the capacity of stem-cell-derived cardiac tissues to self-organize into chamber-like structures^92^. In parallel, iPS cell-derived cardiac microtissues and engineered heart tissues provide multicellular platforms that can incorporate cardiomyocytes, stromal cells, endothelial-like cells, extracellular matrix and mechanical or pharmacological stimulation^93,94^.

These systems are particularly useful for testing hypotheses derived from multi-omics analyses of human tissues. For example, if single-cell or spatial transcriptomics identifies a fibroblast–immune interaction, an endothelial inflammatory niche, a metabolic defect or a stress-response pathway in diseased human myocardium, iPS cell-derived cardiac tissues can be used to perturb that pathway under controlled conditions. Genome editing can generate isogenic controls, while patient-derived iPS cells can preserve disease-associated genetic background. Drug exposure studies, cytokine stimulation, hypoxia–reoxygenation injury, mechanical loading and co-culture with immune or stromal cells can then be used to test whether candidate pathways alter contractility, calcium handling, fibrosis-related gene programs, mitochondrial function, cell death or inflammatory signaling. This provides a practical intermediate step before moving to animal models.

Nevertheless, iPS cell-derived cardiac systems also have important limitations. They often retain immature, fetal-like transcriptional, metabolic and electrophysiological features; may lack the full adult myocardial architecture; incompletely model neurohumoral, vascular, immune and mechanical influences; and remain sensitive to line-to-line and protocol-specific variability. Therefore, they should not be viewed as replacements for human tissue analysis or in vivo validation. Rather, they are best positioned as mechanistic filtering platforms within the bedside-to-bench-and-back framework. Human tissue multi-omics can identify candidate disease mechanisms; iPS cell-derived organoids or engineered tissues can test human-cell-autonomous and multicellular mechanisms under controlled conditions; animal models can evaluate systemic physiology and causality in vivo; and clinical studies can then determine whether the resulting biomarkers or therapeutic targets are relevant to patient care.

### Computational integration, artificial intelligence and biological foundation models

A major challenge in human cardiovascular tissue multi-omics is distinguishing true disease biology from technical variation. This issue is particularly important in studies of human cardiac tissue, where samples may originate from biopsies, surgical specimens, explanted hearts, donor tissues, or autopsies, each associated with different ischemic durations, preservation methods, fixation protocols, tissue-processing pipelines, sequencing platforms and mass spectrometry workflows. In addition, cardiovascular cohorts are often relatively small because access to well-phenotyped human myocardial tissue is limited. Under these conditions, uncorrected batch effects may be mistaken for disease-associated signals, while aggressive correction may remove meaningful biological variation. Therefore, computational integration should be considered a core component of study design rather than a purely downstream analytical step.

Current computational strategies can address these challenges at several complementary levels. First, batch-aware normalization and dataset harmonization methods are essential for integrating single-cell, single-nucleus and spatial datasets generated across patients, centers, and platforms. Methods such as Harmony align shared biological cell states across batches while preserving biologically relevant structure, thereby improving the robustness of cell-state discovery and cross-cohort comparisons^95^. Deep generative models such as single-cell variational inference provide a probabilistic framework for modeling sparse single-cell count data, estimating latent cellular states, accounting for batch effects, and supporting downstream clustering, visualization, differential expression and uncertainty-aware interpretation^96^. These approaches are particularly relevant for human cardiovascular studies, where technical and clinical heterogeneity are often inseparable.

Second, multimodal integration frameworks are increasingly important because cardiovascular disease mechanisms rarely operate within a single molecular layer. Transcriptomic data may identify inflammatory or fibrotic gene programs; proteomics may reveal post-transcriptional remodeling and pathway activation; metabolomics may capture changes in energy and substrate utilization; and epigenomic data may expose regulatory mechanisms underlying cell-state transitions. Weighted-nearest-neighbor approaches allow multiple modalities measured in the same cells to contribute differentially to cellular neighborhood structure, enabling more accurate definition of cell states than any individual modality alone^97^. In parallel, latent-factor models such as MOFA+ can integrate transcriptomic, epigenomic, proteomic and metabolomic datasets across samples or conditions to identify shared and modality-specific molecular programs^98^. In cardiovascular tissue, such methods may help distinguish immune-fibrotic, metabolic, endothelial, electrophysiological and extracellular-matrix remodeling programs and link them to clinical phenotypes.

Third, spatial data integration is essential because the human heart is not molecularly homogeneous. Myocardial inflammation, fibrosis, vascular remodeling, amyloid deposition, ischemic injury and conduction-system niches are spatially organized processes. Bulk RNA sequencing, plasma biomarkers and even dissociated single-cell approaches may lose the anatomical relationships among cardiomyocytes, fibroblasts, endothelial cells, immune cells, the extracellular matrix and vascular structures. Spatial mapping tools such as Tangram can align single-cell or single-nucleus transcriptomic profiles with spatial transcriptomic datasets, enabling inference of where specific cell states and molecular programs are located within tissue architecture^99^. Similarly, cell2location uses a Bayesian framework to map fine-grained cell types in spatial transcriptomic data, supporting the identification of local inflammatory niches, fibrotic regions, vascular microenvironments and multicellular interaction zones^100^. These tools are especially relevant for myocardial infarction, myocarditis, heart failure, atherosclerosis and valvular disease, where pathogenic mechanisms are often compartmentalized within specific tissue regions rather than uniformly distributed.

Fourth, network-level and artificial intelligence-based approaches are needed to move beyond lists of differentially expressed genes, proteins, or metabolites toward interpretable biological mechanisms. Machine learning can support patient stratification, biomarker prioritization, feature selection and prediction of therapeutic response, but these models must be carefully designed to avoid overfitting, particularly in small human tissue cohorts. Robust pipelines should include predefined quality-control steps, batch-effect assessment, dimensionality reduction, appropriate correction for multiple testing, sensitivity analyses, external validation where possible, and biological interpretation at the pathway or network level. Importantly, artificial intelligence models should not be treated as substitutes for mechanistic validation. Rather, they should be used to prioritize candidate pathways, cell states and molecular interactions for subsequent confirmation using orthogonal human cohorts, histology, spatial validation, organoids, engineered heart tissues or animal models.

A rapidly emerging direction is the use of biological foundation models trained on cellular and genomic tokens. These models adapt concepts from large language models by representing genes, cells, perturbations or molecular states as structured biological tokens and by learning contextual relationships from very large single-cell or genomic datasets. Geneformer, for example, uses transfer learning from large-scale single-cell transcriptomic data to predict gene-network behavior and candidate disease regulators in settings where disease-specific datasets are limited^101^. Similarly, scGPT applies generative pretraining to large single-cell datasets and has been proposed for cell-type annotation, batch correction, perturbation prediction and multi-omic integration^102^. In human cardiovascular tissue research, these models may be particularly useful because endomyocardial biopsies, explanted hearts, conduction-system tissues and autopsy samples are rare and often underpowered for conventional machine learning approaches. Foundation models may therefore help transfer information from large public atlases to smaller disease-specific cardiac datasets, enabling improved cell-state annotation, prioritization of regulatory networks, and prediction of candidate perturbations.

Moreover, the use of biological foundation models in cardiovascular research should remain cautious and hypothesis-generating. These models can identify statistical patterns, transfer information across datasets and suggest candidate regulatory networks, but they do not, by themselves, establish causality. Their predictions may also be influenced by biases in the training data, underrepresentation of diseased human cardiac tissues, differences between adult and developmental cell states, and platform-specific technical artifacts. Therefore, foundation-model predictions should be interpreted as prioritization tools rather than definitive mechanistic evidence. The strongest applications of these approaches will probably involve integration with spatial localization, clinical metadata, histopathology and experimental validation. In this way, computational integration and artificial intelligence can help convert complex human tissue multi-omics into reproducible disease endotypes, biologically coherent networks, clinically useful biomarkers and experimentally testable therapeutic hypotheses.

### Liquid biopsies and tissue-informed circulating biomarkers

A major future direction for human-first cardiovascular discovery is translating tissue-level molecular signatures into non-invasive biomarkers. Although myocardial, valvular, vascular and autopsy-derived tissues provide the most direct view of disease biology, their limited availability prevents repeated sampling in routine clinical practice. Liquid biopsy approaches may help bridge this gap by capturing circulating molecular signals that reflect tissue injury, cell death, inflammation, fibrosis, endothelial dysfunction, metabolic stress or intercellular communication.

Cell-free DNA is one promising modality because methylation patterns can be used to infer tissue or cell type of origin. Cardiomyocyte-specific methylation signatures in circulating cell-free DNA have been developed as a non-invasive approach to detect human cardiomyocyte death^103^. In cardio-oncology, circulating cardiomyocyte cell-free DNA has also been associated with cancer therapy-related cardiac dysfunction, suggesting a potential role for early detection of subclinical myocardial injury before overt systolic dysfunction develops^104^. These approaches are conceptually important because they attempt to move beyond conventional injury biomarkers by identifying the tissue source of circulating molecular injury signals.

Circulating microRNAs, cell-free RNA and extracellular vesicle-associated cargo represent complementary liquid biopsy strategies. Unlike cell-free DNA, which mainly reflects cell death or tissue injury, circulating RNAs and extracellular vesicles may capture active stress responses, immune activation, fibroblast signaling, endothelial dysfunction and metabolic remodeling. Candidate microRNAs have been studied as diagnostic and prognostic biomarkers in heart failure and ischemic heart disease, although results remain heterogeneous because of differences in patient populations, sample processing, RNA isolation, normalization strategies, analytical platforms and clinical endpoints^105,106^. Extracellular vesicles may confer additional biological specificity by carrying proteins, lipids and regulatory RNAs derived from cardiomyocytes, endothelial cells, fibroblasts, immune cells or extracardiac organs^107^.

The most rigorous strategy is to integrate liquid biopsy measurements with tissue-defined molecular programs. First, human tissue multi-omics can identify disease-relevant modules, including fibroblast activation, immune-cell recruitment, endothelial inflammation, mitochondrial dysfunction, extracellular-matrix remodeling and cardiomyocyte stress. Second, candidate circulating markers can be selected from molecules linked to these tissue-defined pathways. Third, these candidates should be tested in matched tissue–blood cohorts to determine whether circulating levels reflect local tissue abundance, spatial localization, disease stage and clinical phenotype. Finally, longitudinal validation is required to determine whether these markers predict disease onset, progression, therapeutic response or adverse clinical outcomes.

This approach may be particularly useful in diseases where repeated tissue sampling is impractical, including HFpEF, myocarditis, ischemic remodeling, aortic stenosis, cardiac amyloidosis and cardio-oncology. In HFpEF, tissue-informed blood biomarkers may help to distinguish inflammatory-fibrotic, metabolic, endothelial or renal-systemic phenotypes. In myocarditis, liquid biopsies may support monitoring when biopsy is not performed or when sampling error is likely. In cardio-oncology, cardiomyocyte-derived cell-free DNA or extracellular vesicle-associated RNA signatures may help to identify early myocardial injury before clinical heart failure develops. Thus, liquid biopsy approaches may complete the translational loop by converting invasive tissue discoveries into scalable tools for early diagnosis, risk stratification, treatment monitoring and patient selection.

## Conclusion

Taken together, the studies reviewed here show that direct molecular analysis of human cardiovascular tissues can reveal disease mechanisms that are often difficult to identify in animal models alone. In this context, we suggest reversing the traditional research paradigm, moving from a conventional ‘bench-to-bedside’ approach to a ‘bedside-to-bench-and-back’ model (see visual abstract). We envision a research process that begins with human tissues and uses advanced multi-omics technologies to identify key molecular drivers of disease. This human-centered discovery phase would then guide targeted validation in animal models, focusing only on the most important molecular targets. Ultimately, the most promising findings could be translated back into human clinical research. This integrated strategy may streamline scientific work, reduce reliance on animal testing, improve ethical standards and accelerate cardiovascular innovation in the twenty-first century. The examples reviewed above illustrate that the most productive translational pathway is iterative: patient-derived molecular discovery should generate candidate mechanisms, experimental systems should establish causality, and clinical studies should then evaluate whether these mechanisms can improve diagnosis, stratification or therapy.

The rationale for integrating omics into cardiovascular research has been articulated by Mulvey and colleagues, who emphasized that genomics, proteomics and metabolomics provide complementary insights into inherited risk, disease mechanisms, biomarker discovery and therapeutic target identification^108^. In the human-first framework proposed here, these technologies are most powerful when anchored in clinically derived cardiovascular tissues and linked to experimental validation.

As demonstrated throughout this Review and summarized in Table 2, the growing availability of human cardiovascular tissues, biobanks, surgical specimens, post-mortem donations, organoid platforms and high-resolution molecular technologies is reshaping translational cardiovascular research. The broader literature supporting this shift is presented in Supplementary Table 1. By anchoring target discovery in human disease biology and then testing causality in experimental systems, the human-to-animal-to-human loop may improve the precision and efficiency of cardiovascular drug development.

This reverse translational model also requires robust ethical and procedural standards for tissue acquisition, consent, data sharing and early-phase human research. With appropriate safeguards, a human-first approach may improve translational fidelity while preserving rigorous ethical oversight.

## Supplementary information

Supplementary Information
