# Supplementary material for: Reversing the pipeline: a ‘human-first’ multi-omics approach to cardiovascular discovery
Source: Exp Mol Med. 2026 Sep 9;58(9):2859–72. doi: 10.1038/s12276-026-01835-8 (PMC13633249; doi:10.1038/s12276-026-01835-8)
Supplement: Supplementary file 1 [file 12276_2026_1835_MOESM1_ESM.pdf]

Supplementary Table 1. Comprehensive evidence table of cardiovascular human tissue and translational multi-omics studies. This table provides the complete list of studies reviewed in the manuscript, including disease area, study population or model, tissue source, molecular method, and key findings.

Section	Study	Tissue Source	The purpose (Population/ Model)	Methods	Key Findings
**Heart Failure**	Hahn VS. et al. 2021 (2)	Human cardiac tissue	HFpEF	RNA sequencing on right ventricular septal endomyocardial biopsies	HFpEF shows a distinct transcriptomic profile.
	Hahn VS. et al. 2021 (3)	Human myocardial tissue and plasma	HFpEF vs HFrEF vs non-failing donor	Targeted metabolomics on myocardium and plasma	HFpEF myocardium exhibits reduced fatty acid oxidation and impaired TCA cycle metabolism, indicating metabolic inflexibility.
	Lopez B. et al. 2006 (4)	Human cardiac tissue	Diastolic heart failure (DHF) vs Systolic heart failure (SHF)	Western blot and immunohistochemistry for tissue, enzyme-linked immunosorbent assay for blood.	SHF hypertensive patients show increased MMP-1 activity with excessive collagen degradation, distinguishing them from DHF and linking to impaired systolic function.
	Querejeta R et al 2004 (5)	Human cardiac tissue	HF vs NON-HF	Histomorphological and Immunohistochemical analysis of tissues. And radioimmunoassay of serum samples	Excess collagen type I synthesis and deposition drive myocardial fibrosis in hypertensive heart disease, with elevated coronary PICP secretion making peripheral PIP a marker of fibrosis and HF progression.
	Fayyaz A. U. et al. 2023 (6)	Human autopsy specimens	HF-related PH vs Controls	Laser-capture microdissection of pulmonary arteries and veins; proteomics and phosphor-proteomics.	Pulmonary arterial and venous remodeling showed proteomic signatures that overlapped with the porcine model, supporting conserved stress and signaling pathways.
	Litviňuková M. et al 2020 (9)	Human cardiac tissue	human cardiac cell atlas	single-cell and single-nucleus RNA sequencing	Generated a multi-chamber adult human heart atlas that reveals cardiac cellular diversity, chamber-specific cell states, and rare populations, serving as a reference for disease interpretation.

	Bishu K. et al. 2011 (13)	Dog cardiac tissue	Elderly hypertensive dogs versus young control dogs.	Hemodynamic assessment and measurement of the end-diastolic pressure-volume relationship.	Sildenafil and B-type natriuretic peptide enhanced cGMP signaling, increased titin phosphorylation, reduced cardiomyocyte stiffness, and improved left ventricular diastolic distensibility.
	Takimoto E. et al. 2005 (14)	Mice cardiac tissue	Mice undergo transverse aortic constriction (TAC) or sham operation.	Cardiac function evaluation using echocardiogram, Histology, Western blot,	Sildenafil inhibits phosphodiesterase-5A, enhances cGMP signaling, and prevents or reverses pressure overload-induced cardiac hypertrophy while restoring function.
	Bermejo J. et al. 2018 (15)	Human double-blind, randomized clinical trial	Patient outcomes were compared between those who received sildenafil and those who received a placebo.	Composite clinical score combining: death, hospital admission for heart failure (HF), change in functional class, and patient global self-assessment.	In patients with persistent pulmonary hypertension after corrected valvular heart disease, sildenafil worsened clinical outcomes compared with placebo, indicating it should not be used off-label in this setting.
	Wang L, et al. 2020 (21)	Human cardiac tissue	Failing and LVAD-supported human hearts.	Single-nucleus RNA sequencing	Single-cell analysis revealed dynamic changes in cardiomyocytes, endothelial cells, fibroblasts, macrophages, and their subtypes during heart failure and mechanical unloading.
	Chaffin et al. 2022 (22)	Human cardiac tissue	DCM vs HCM vs Controls	Single-nucleus RNA sequencing and CRISPR-mediated functional validation.	Identified disease-associated fibroblast states and candidate regulators of myofibroblast differentiation.
	Tan et al. 2025 (23)	Human cardiac tissue	Early-stage DCM	Epigenome-wide DNA methylation analysis.	CpG methylation changes predicted progression to overt heart failure, supporting potential epigenetic biomarkers of early cardiac risk.
	Perry et al. 2024 (24)	Blood and myocardial tissue correlation.	HF (general)	Plasma proteomics integrated with myocardial transcriptomic signatures and echocardiographic phenotypes.	Identified circulating proteins linked to myocardial remodeling signatures, supporting tissue-informed biomarker discovery.
	Drakos et al. 2023 (25)	Human cardiac tissue	HF with LVAD vs without LVAD	RNA-seq, phospho-proteomics	Signatures distinguish responders vs. non-responders; ECM pathways.
	Hahn VS et al. 2025 (26)	Human cardiac tissue	HFpEF vs Healthy controls	Genotype-based demultiplexing and gene expression quantified with CellRanger and CellBender. limma-voom and functional analysis performed	Single-nucleus RNA sequencing shows that HFpEF is driven primarily by cardiomyocyte transcriptional changes, with distinct pathways that separate it from dilated cardiomyopathy.

				using Gene Set Enrichment Analysis	
	Hahn VS et al. 2024 (27)	Human cardiac tissue	41 patients with HFpEF.	NPPB gene transcriptome	In HFpEF, obesity lowers myocardial NPPB synthesis and serum NT-proBNP despite similar hemodynamics, challenging current diagnostic thresholds.
	Hahn VS et al. 2023 (28)	Human myocardial tissue and plasma.	HFpPF vs HFrEF vs non-failing donor controls	Quantitative targeted metabolomics	In HFpEF, myocardial metabolomics revealed reduced fatty acid, ketone, TCA cycle, and BCAA metabolites despite severe obesity and diabetes, indicating marked fuel inflexibility not detectable in plasma.
	Hahn VS et al. 2021 (29)	Human cardiac tissue	HFpPF vs HFrEF vs non-failing donor controls	RNA sequencing	RNA sequencing of myocardial biopsies showed HFpEF has distinct transcriptomic signatures compared with HFrEF and controls, marked by mitochondrial upregulation, downregulation of stress and angiogenesis pathways, and three molecular subgroups with differing clinical profiles and outcomes.
	Amrute JM et al. 2024 (30)	Human cardiac tissue	6 Healthy donors vs four acute MI patients vs six ischemic cardiomyopathy patients vs six non-ischemic cardiomyopathy patients	Multi-omic single-cell gene expression, epitope mapping, and chromatin accessibility profiling	Single-cell multi-omics of human hearts revealed a fibroblast trajectory leading to FAP/POSTN fibroblasts driven by IL-1β signaling from CCR2+ macrophages; blocking this pathway reduced fibrosis and improved function, identifying a therapeutic target for cardiac disease.
	Borger J et al. 2025 (31)	Human cardiac tissue	44 patients with ischemic cardiomyopathy and 37 with dilated cardiomyopathy undergoing LVAD (59) or heart transplantation (22)	Quantification of mitochondrial oxidative capacity, citrate synthase activity (CSA), fibrosis, and lymphocytic infiltration.	In advanced heart failure, ischemic cardiomyopathy shows greater inflammation and reduced mitochondrial oxidative capacity than dilated cardiomyopathy.
	Rao M. et al. 2021 (32)	Human cardiac tissue	Dilated cardiomyopathy (DCM) and Ischemic cardiomyopathy (ICM)	single-cell RNA sequencing and single T cell receptor sequencing	Single-cell analyses of human DCM and ICM hearts revealed AEBP1 as a key regulator of cardiac fibrosis, with fibrotic lesions enriched in pro-inflammatory T cells and $CXCL8^{+}CCR2^{+}$ macrophages that interact with endothelial cells to drive leukocyte recruitment.
	Lumish HS et al. 2024 (33)	Human cardiac tissue	Hypertrophic cardiomyopathy (HCM) vs Control subjects with nonfailing hearts.	Proteomic profiling using the SomaScan assay (SomaLogic)	Proteomic and transcriptomic profiling in HCM revealed dysregulated Ras-MAPK, angiogenesis, Hippo, and metabolic-inflammatory pathways linked to disease progression.

	Tan WLW et al. 2020 (34)	Human cardiac tissue	Human control and end-stage failing hearts.	Epigenomic profiling of enhancer H3K27ac and QTL analysis.	Mapped cardiac enhancers and histone-acetylation QTLs, linking regulatory variation to gene expression and heart failure risk.
	Junttila MJ et al. 2025 (35)	Human cardiac tissue	Sudden cardiac death with primary myocardial fibrosis.	Targeted next-generation sequencing using a panel of 174 genes associated with myocardial structure and ion channel function	Postmortem sequencing revealed cardiomyopathy-related variants in many young sudden cardiac death cases with primary myocardial fibrosis, suggesting PMF may represent an early or alternative manifestation of genetic heart disease with implications for family risk assessment.
**Ischemic Heart Disease**	Sandstedt et al. 2023 (37)	Myocardium (CABG, AVR)	To map the presence of microbes in the myocardium of patients with heart disease	Metagenomic sequencing and Toll-like receptor pathway analysis.	Metagenomic sequencing detected microbial RNA in human cardiac tissue and linked microbial signatures to Toll-like receptor-associated inflammation.
	Kuppe et al. 2022 (38)	Post-MI myocardium	Post-myocardial infarction remodeling.	Single-nucleus RNA sequencing, ATAC-seq, and spatial transcriptomics.	Mapped post-infarction remodeling in spatial context, identifying cell-type-specific programs and microenvironmental signaling across infarct and border-zone regions.
	Linna-Kuosmanen et al. 2024 (39)	Human right atrial tissue	Ischemic remodeling	Transcriptomics and spatial analysis	Identified microvascular inflammation and metabolic reprogramming associated with ischemic remodeling and the development of heart failure.
	Gastanadui M.G et al. 2024 (40)	Autopsy coronary plaque	Plaque stability	Spatial transcriptomics and histological analysis.	Spatial analysis of human coronary plaques identified inflammatory activity and smooth-muscle-cell-derived phenotypes associated with plaque instability.
	Bashore AC et al. 2024 (41)	Human carotid atherosclerotic plaques	To understand the comprehensive transcriptional and phenotypical landscape of the cells within the lesions.	CITE-seq and single-cell RNA sequencing	Single-cell and CITE-seq mapping of human carotid plaques revealed 25 cell populations and enrichment of inflammatory smooth muscle cells in symptomatic lesions, highlighting new therapeutic targets.
	Fernandez DM et al. 2019 (42)	Human carotid artery plaques	To identify specific characteristics of dysregulated immune cells within atherosclerotic lesions that lead to clinical events such as ischemic stroke or myocardial infarction	Single-cell proteomic and transcriptomic analyses	Symptomatic carotid plaques contain exhausted T cells and vulnerable macrophages, while asymptomatic plaques show IL-1β-driven immune activation.

	Depuydt MAC et al. 2020 (43)	human carotid atherosclerotic plaques	To define cells and to determine their transcriptomic and epigenomic characteristic inside the carotid atherosclerotic plaques	single-cell transcriptomics and chromatin accessibility	Single-cell transcriptomics of human plaques revealed 14 cell types with dynamic states, including inflammatory macrophages and endothelial-to-mesenchymal transition, highlighting key targets in atherosclerosis.
	Tokcan et al. 2024 (44)	Coronary arteries (donors)	To quantitatively examine periarterial innervation in human epicardial coronary arteries.	Histology, immunofluorescence	Mapped 42,573 nerve fibers; regional sympathetic patterns.
	Shami et al. 2020 (45)	Human vascular tissue	To check the relevance of GITR in cardiovascular disease (CVD).	RNA sequencing	GITR expression and elevated sGITR levels were associated with plaque instability and increased cardiovascular risk.
	Bleckwehl T et al. 2025 (46)	Human atherosclerotic plaques	To integrate a high-resolution map of human atherosclerotic plaques	Single-cell RNA sequencing from multiple studies and spatial transcriptomics data from 12 human specimens with different stages of atherosclerosis.	Integrated single-cell and spatial mapping of human plaques uncovered stage-specific gene changes, lymphocyte recruitment, smooth muscle transition, and complex cellular interactions in atherosclerosis.
**Electrophysiology**	Gelband H et al. 1972 (47)	Human cardiac tissue	Electrophysiology of the heart	Intracellular recordings using glass microelectrodes from human right atrial tissue.	Characterized human atrial action potentials ex vivo.
	Trautwein W. et al. 1962 (48)	Human cardiac tissue	Compared atrial and ventricular action potentials	Intracellular microelectrode recordings from human heart tissue.	Identified distinct atrial and ventricular action potential profiles.
	Kanemaru et al. 2023 (49)	Conduction tissue, pericardium	Conduction physiology	Spatial transcriptomics, single-cell analysis, and histology.	Spatial multi-omics mapped specialized human cardiac niches, including fibroblast-glial interactions and immune microenvironments within conduction and pericardial tissues.
	Vyas et al. 2024 (50)	EAT from surgery patients	To investigate how the immune infiltrate of human epicardial adipose tissue (EAT), which directly overlies the myocardium, contributes to atrial fibrillation.	CITE-seq (CITE-seq) and single-cell T cell receptor (TCR) sequencing. And transcriptomic analysis of EAT and atrial tissue	Epicardial adipose tissue in AF harbors enriched $CD8^{+}$ tissue-resident memory T cells that drive inflammation, fibrosis, and cardiomyocyte dysfunction, promoting arrhythmia vulnerability.

	Leblanc FJA et al. 2025 (52)	Left atrium tissue from atrial fibrillation patients	To understand the dysregulation of gene expression programs in the human atria during persistent atrial fibrillation (AF).	Single-nucleus multi-omics.	Persistent AF was associated with androgen-receptor signaling in cardiomyocytes, immune remodeling, and NR4A1-related fibroblast activation.
**Myocarditis**	Callon D et al. 2024 (53)	Autopsy human cardiac tissue	Genetic cardiomyopathies and viral myocarditis	Histology, PCR, and Next-Generation Sequencing	Linked viral myocarditis and underlying genetic cardiomyopathy to sudden cardiac death.
	Kloc A et al. 2023 (54)	Human cardiac tissue (Biopsy or post-mortem)	To identify viral genomes in human heart tissues for disease diagnosis and assessment of cardiovascular damage.	Total DNA was isolated from the heart tissue under varying conditions. A PCR-based assay with Parvovirus B19-specific primers was implemented to detect the presence of the viral genome, followed by Sanger Sequencing.	PCR-based detection of Parvovirus B19 in human heart tissue showed that DNA yield and viral genome identification depend strongly on tissue disruption and processing methods.
	Sobol I et al. 2020 (55)	EMB human myocardium	To review the histology of myocardial biopsy-proven cases of immune checkpoint-associated myocarditis and provide immunohistochemical characterization of the inflammatory infiltrate.	Histological staining techniques	Checkpoint inhibitor-associated myocarditis shows diffuse infiltrates of $CD163^+$ macrophages and $CD8^+$/$PD\text{-}1^+$ T cells, with PD-L1-positive myocytes.
	Pang et al. 2024 (56)	EMB human myocardium	ICI-induced myocarditis vs non-ICI troponemia (elevated troponins from non-immune etiology) vs Controls matched by tumor type, with no IRAEs nor troponemia.	multi-omics pipeline for ICI-myocarditis, integrating scRNA/TCR/BCR sequencing of PBMCs with DNA methylation, cytokine profiling, and spatial proteomics to map cytokine sources and cell-cell interactions driving pathology.	Revealed T cell/macrophage infiltration and elevation of IL signaling.
	Sun B. et al. 2025 (57)	Human cardiac tissue	To elucidate the inflammatory microenvironment within cardiac tissues affected by immune-related myocarditis at the single-cell level to identify potential therapeutic targets.	Single-cell RNA sequencing (scRNA-seq) on an endomyocardial biopsy specimen obtained from a patient with pancreatic neuroendocrine carcinoma who developed immune-related myocarditis following treatment with ICIs.	ICI-myocarditis features exhausted $CD8^+$ T cells, pro-inflammatory fibroblasts, and $ACKR1^+$ endothelial interactions, suggesting new therapeutic targets.

	Maatz H et al. 2025 (58)	Human cardiac tissue	To understand the molecular mechanisms of myocarditis after severe acute respiratory syndrome coronavirus 2 (SARS-CoV-2) infection or mRNA-based coronavirus disease 2019 (COVID-19) vaccination	Single-nucleus RNA sequencing on the left ventricle	Single-nucleus RNA sequencing revealed distinct immune signatures in myocarditis: IFN-γ-driven in post-COVID-19, IL16/IL18-driven in post-vaccination, with differing T-cell subsets and endothelial dysfunction shaping disease mechanisms.
	Blum SM et al. 2024 (59)	Human cardiac tissue	To understand the pathogenesis of irMyocarditis and its relationship to antitumor immunity.	single-cell RNA sequencing coupled with T cell receptor (TCR) sequencing, microscopy, and proteomics analyses	IrMyocarditis is marked by cytotoxic T cells, dendritic-fibroblast interactions, immune shifts in the blood, and fatality-linked TCR clones.
**Cardio-oncology**	Udin MH et al. 2024 (61)	Autopsy tissue	Hematologic vs. solid tumors	Histopathology, SHG imaging, immunohistochemistry, and serum biomarker analysis.	Greater collagen disruption and biomarker elevation in hematologic cancer.
	Kushnareva E, et al. 2022 (62)	Human cardiac tissue	Anti-PD-L1 antibody treatment induces cardiac toxicity	Immunohistochemistry, H&E staining, and correlation with echocardiographic data.	Increased myocardial PD-L1 expression correlates with worse cardiac function.
	Nguyen N et al. 2021 (63)	In vitro human cardiac microtissues (tissue collected by heart biopsy)	To study the proteome-level effects of 3 common antitumor drugs known to be cardiotoxic.	Proteomic alterations in cardiac tissues caused by three anthracycline analogs: doxorubicin, idarubicin, and epirubicin,	Proteomic analysis of anthracycline-treated cardiac tissues identified 11 key proteins, primarily associated with mitochondrial function and NF-κB signaling, highlighting potential biomarkers and therapeutic targets for anthracycline-induced cardiotoxicity.
**Amyloidosis**	Kourelis et al. 2020 (67)	Cardiac amyloid plaques	ATTR vs. AL	Proteomics	Identified 28 ATTR/19 AL-enriched proteins that exhibit distinct stress responses.
	Netzel BC et al. 2025 (68)	Human cardiac tissue	ATTR, AL - staging	Proteomics, transcriptomics	Stage-specific ECM and immune alterations; ATTR: complement up. AL: endocytic and ribosomal signatures.
	Sukhacheva TV et al. 2021 (69)	Human Atrial Appendage	To determine the ultrastructural and immunohistochemical features of cardiac telocytes in patients with atrial fibrillation (AF) and in	Immunohistochemistry and double immunofluorescence techniques	Increased telocytes and CD68+ cells in combined disease; role in atrial remodeling.

			those with AF + isolated atrial amyloidosis.		
	Cimini M et al. 2025 (70)	Mice and Human cardiac tissue	To explore the relations between amyloidosis and myocardial infarction.	Flow cytometry, immunohistochemistry analysis, single-cell RNA sequencing, and proteomics analysis using mass spectrometry	Post-MI exosomal signaling drives SAA3 amyloid deposition, while DRI-R5S blocks aggregation and preserves function.
	Noborn F et al. 2023 (71)	Human cardiac tissue	To introduce a direct proteomics-based method for subtyping of cardiac amyloidosis.	Cryostat sections were digested with trypsin and analyzed by liquid chromatography-mass spectrometry, and the data were evaluated using proteomic software.	Mass spectrometry of endomyocardial biopsies enabled accurate subtyping of cardiac amyloidosis without laser microdissection, achieving 94% concordance with original diagnoses.
**Aortic Stenosis**	Blankenberg S et al. 2024 (72)	Not applicable; clinical trial.	Severe aortic stenosis: TAVI versus SAVR.	Randomized controlled trial	In low- or intermediate-risk patients with severe aortic stenosis, TAVI was noninferior to SAVR at 1 year, with lower rates of death and stroke.
	Martín-Rojas et al. 2012 (74)	Human aortic valve tissue	Aortic stenosis vs Normal aortic tissue	Two-dimensional difference gel electrophoresis and mass spectrometry.	Identified altered contractile, inflammatory, oxidative-stress, and metabolic proteins in calcified human aortic valves.
	Schlotter F et al. 2021 (75)	Human aortic valve tissue	To characterize the human AV apolipoprotein distribution across different Calcific aortic valve disease tissues	Proteomics and immunohistochemistry	Proteomic and histologic analyses of calcified valves revealed multiple apolipoproteins enriched in diseased tissue, with apoC-III not only marking but actively driving valvular calcification through mitochondrial dysfunction and inflammation.
	Schlotter F et al. 2018 (76)	Human aortic valve tissue	To identify the first molecular regulatory networks in Calcific aortic valve disease (CAVD) tissues	Transcriptomics and global, unlabeled and label-based tandem-mass-tagged proteomics, near-infrared molecular imaging, and anatomic layer specificity were facilitated by laser capture microdissection and cell cultures.	Multi-omics and spatial proteogenomic mapping of human stenotic valves revealed stage- and layer-specific molecular signatures, identified interstitial cell subtypes with distinct calcification potential, and uncovered regulatory networks linking CAVD to inflammatory and fibrotic diseases, offering new therapeutic targets.
	Sáinz-Jaspeado M et al. 2021 (77)	Human aortic valve tissue	To provide a mechanistic basis for PALMD's (palmdelphin) contribution to the pathology of calcific aortic valve stenosis.	Mass spectrometry, coimmunoprecipitation, and immunofluorescence. RNA sequencing of ECs and transcript arrays.	PALMD loss impairs nucleocytoplasmic transport and nuclear mechanics, driving calcific aortic valve stenosis.

	Brandenburg S et al. 2022 (78)	Human Myocardium and the Aortic Valve Tissues.	Aortic Stenosis (AS) - various EF/gradient profiles	Proteomics- mass spectrometry (DIA-MS)	Proteomic and microscopy analyses of AS patient biopsies revealed subtype-specific myocardial proteotypes, with RyR2 disruption and t-tubule uncoupling linked to systolic dysfunction, providing mechanistic insight into divergent AS outcomes.
	Blaser MC et al. 2023 (79)	Human carotid plaques + aortic valves	calcific aortic valve stenosis vs. atherosclerosis	Vesiculomics, comprising vesicular proteomics and small RNA-sequencing	EVs in plaques and valves carry distinct calcification drivers: Notch signaling in arteries and Wnt signaling in valves. Their tissue-specific cargoes actively modulate disease progression. Vesiculomics reveals novel mechanisms underlying cardiovascular calcification.
	Bourgeois et al. 2021 (80)	Human aortic valve and blood tissues	To understand the mechanisms through which Lp(a) causes calcific aortic valve stenosis	Proteomics and transcriptomic signature of calcified aortic valves.	Multi-omic analysis linked Lp(a)-associated calcific aortic valve disease to inflammatory, platelet-activation, wound-response, aging, and chondrocyte-development pathways.
	Pal N et al. 2024 (81)	Human cardiac tissue	Aortic stenosis- associated left ventricular hypertrophy versus hypertrophic cardiomyopathy.	Metabolomic and orthogonal transcriptomic analysis	Invasive metabolomic and transcriptomic profiling revealed progressive impairment of β-oxidation from HCM to severe AS, with long-chain acylcarnitine accumulation and downregulated PPAR-α signaling, highlighting disrupted fatty acid oxidation as a therapeutic target in AS.
	Heuschkel et al. 2020 (82)	Human calcified aortic valves	To perform integration of multiple omics data for unveiling novel molecular networks in Calcific aortic valve disease	Multi-omics	Multi-omics integration in CAVD revealed dysregulated platelet and complement pathways, a network of key genes and miRNAs, and links to amyloid-related proteins (APP, TTR), suggesting amyloid-like deposits as novel contributors and therapeutic targets in valve calcification.
	Fu B et al. 2022 (83)	Human calcific aortic valve tissue.	To define changes in the metabolic and protein profiles of patients with calcific aortic valve disease	Metabolomics and tandem mass tag-based quantitative proteomics.	Integrated metabolomic and proteomic profiling of CAVD valves revealed 229 altered metabolites and 549 proteins, highlighting pathways such as HIF-1 and purine metabolism, with hub proteins ENPP1, CAMK2D, and ABCA8 emerging as potential biomarkers and therapeutic targets.
	Antequera-González B et al. 2024 (84)	Human aortic valve with tricuspid valve and bicuspid valve	To identify differences in the pathophysiology of AS between the bicuspid aortic valve and the tricuspid aortic valve	Multi-omics	Multi-omics profiling revealed subtype-specific RNA and metabolic signatures, including mitochondrial dysfunction and oxidative stress in bicuspid valve disease.
	Guauque-Olarte S et al. 2016 (85)	Human aortic valve with and without a calcified tricuspid valve and a calcified bicuspid valve	To identify genes differentially expressed between calcified bicuspid aortic valves (BAVc) and tricuspid valves with (TAVc) and without (TAVn) Aortic stenosis.	RNA sequencing	RNA sequencing revealed that bicuspid and tricuspid calcified valves exhibit similar gene expression changes in AS. However, BAV displays additional alterations, particularly in fibrosis-related pathways, indicating enhanced remodeling as a key mechanism and a potential therapeutic target.

	Decano JL et al. 2022 (86)	Human calcific aortic valve tissue.	To identify a disease-driver population (DDP) within valvular interstitial cells (VICs)	Single-cell analysis, phenotype-guided omics profiling, and network-based analysis	Single-cell and multi-omics analyses identified a disease-driver VIC population in CAVD with osteogenic potential, defined by CD44^highCD29^+CD59^+CD73^+CD45^low, and revealed MAOA and CTHRC1 as therapeutic targets.
	Thériault S et al. 2024 (87)	Human Valve tissue	To identify novel molecular targets for medical therapy to prevent calcific aortic valve stenosis	RNA sequencing	A large GWAS meta-analysis of CAVS identified 32 risk loci (20 novel) and prioritized causal genes, including TWIST1, with transcriptomic and integrative analyses implicating lipoproteins, blood pressure, and inflammation as key drivers and therapeutic targets.
	Barbarics B et al. 2021 (88)	Formalin-fixed and paraffin-embedded (FFPE) human myocardial tissue from patients with aortic stenosis.	To understand the proteome-level myocardial changes in hearts with aortic valve stenosis.	High-resolution mass spectrometry in combination with a stem cell-derived cardiac-specific protein quantification standard.	High-resolution proteomics of FFPE myocardial tissue from AVS patients quantified ~2000 proteins per sample, revealing atrial and ventricular changes characterized by hypertrophy, fibrosis, mitochondrial impairment, and reduced blood supply, providing a new proteomic resource for AVS pathology.

Abbreviations: 2D-DIGE, two-dimensional difference gel electrophoresis; AA, amyloid A; ABCA8, ATP-binding cassette subfamily A member 8; ACKR1, atypical chemokine receptor 1; AF, atrial fibrillation; AL, light chain amyloidosis; AS, aortic stenosis; ATAC-seq, assay for transposase-accessible chromatin using sequencing; ATTR, transthyretin amyloidosis; BAV, bicuspid aortic valve; BCAA, branched-chain amino acid; BNP, B-type natriuretic peptide; CABG, coronary artery bypass grafting; CAVD, calcific aortic valve disease; CCR2, C-C chemokine receptor type 2; CD, cluster of differentiation; cGMP, cyclic guanosine monophosphate; CSA, citrate synthase activity; CSC, cardiac stromal cell; CTHRC1, collagen triple helix repeat containing 1; CXCL, C-X-C motif chemokine ligand; DARC, Duffy antigen receptor for chemokines; DCM, dilated cardiomyopathy; DHF, diastolic heart failure; EAT, epicardial adipose tissue; EBV, Epstein-Barr virus; EF, ejection fraction; EV, extracellular vesicle; GITR, glucocorticoid-induced TNFR-related protein; GLP-1, glucagon-like peptide 1; GWAS, genome-wide association study; H3K27ac, acetylation of histone H3 at lysine 27; haQTL, histone acetylation quantitative trait locus; HCM, hypertrophic cardiomyopathy; HF, heart failure; HFpEF, heart failure with preserved ejection fraction; HFrEF, heart failure with reduced ejection fraction; HHV6, human herpesvirus 6; HIF-1, hypoxia-inducible factor 1; HLA, human leukocyte antigen; ICI, immune checkpoint inhibitor; ICM, ischemic cardiomyopathy; Ig, immunoglobulin; IHD, ischemic heart disease; IL, interleukin; LAD, left anterior descending artery; LC-MS, liquid chromatography-mass spectrometry; LCMD, laser capture microdissection; LCx, left circumflex artery; LMCA, left main coronary artery; LVAD, left ventricular assist device; LVEDV, left ventricular end-diastolic volume; LVEF, left ventricular ejection fraction; LVH, left ventricular hypertrophy; MAOA, monoamine oxidase A; MI, myocardial infarction; MMP, matrix metalloproteinase; NK, natural killer; NT-proBNP, N-terminal pro-B-type natriuretic peptide; PA, pulmonary artery; PALMD, palmdelphin; PCR, polymerase chain reaction; PD-1, programmed cell death protein 1; PD-L1, programmed death-ligand 1; PDPN, podoplanin; PH, pulmonary hypertension; PIP, procollagen type I C-terminal propeptide; PMF, primary myocardial fibrosis; PPAR, peroxisome proliferator-activated receptor; PV, pulmonary vein; RCA, right coronary artery; RNA, ribonucleic acid; RyR2, ryanodine receptor 2; SAA3, serum amyloid A3; SAVR, surgical aortic valve replacement; SHF, systolic heart failure; SMC, smooth muscle cell; TAV, tricuspid aortic valve; TAVR, transcatheter aortic valve replacement; TCA, tricarboxylic acid; TCR, T cell receptor; TGF, transforming growth factor; TLR, Toll-like receptor; TMAO, trimethylamine N-oxide; TNF, tumor necrosis factor; TREM2, triggering receptor expressed on myeloid cells 2; TRM, tissue-resident memory T; VEGF, vascular endothelial growth factor; WGCNA, weighted gene co-expression network analysis.
